# Impact of Lid Wipes and Tear Substitutes on Symptoms and Signs of Ocular Surface Disease After Cataract Surgery—A Real-Life Study

**DOI:** 10.3390/jcm14228140

**Published:** 2025-11-17

**Authors:** Giulia Coco, Laura Antonia Meliante, Francesca Di Stefano, Livio Vitiello, Giuseppe Giannaccare

**Affiliations:** 1 Ophthalmology Unit, Department of Clinical Sciences and Translational Medicine, University of Rome Tor Vergata, 00133 Rome, Italy; giulia.coco@uniroma2.it (G.C.); lauraantonia.meliante@ptvonline.it (L.A.M.);; 2 Eye Unit, “Luigi Curto” Hospital, Azienda Sanitaria Locale Salerno, 84035 Polla, Italy; livio.vitiello@gmail.com; 3 Eye Clinic, Department of Surgical Sciences, University of Cagliari, Via Università 40, 09124 Cagliari, Italy

**Keywords:** dry eye disease, ocular surface disease, cataract, tear substitutes, eyelid hygiene

## Abstract

**Objective:** To evaluate the efficacy of lid wipes and tear substitutes, in addition to standard postoperative treatment, in alleviating signs and symptoms of ocular surface disease (OSD) following cataract surgery. **Methods:** Retrospective study on patients who underwent cataract surgery and received either standard postoperative treatment (topical antibiotics, corticosteroids, and nonsteroidal anti-inflammatory drugs) or the same regimen supplemented with lid wipes and tear substitutes. Preoperatively and one month postoperatively, symptoms were evaluated using the 5-item Dry Eye Questionnaire (DEQ-5) and the Ocular Surface Disease Index (OSDI), while noninvasive keratograph break-up time (NIKBUT), tear meniscus height (TMH), conjunctival hyperemia, and infrared meibography were measured by the Oculus Keratograph. **Results:** A total of 63 patients (mean age 75.1 ± 6.3 years) were analyzed. Patients receiving standard treatment showed no significant changes in OSDI (+2 ± 32.7; *p* = 0.859) or DEQ-5 scores (+1.7 ± 5.4; *p* = 0.204). Conversely, those receiving the adjunct of lid wipes and tear substitutes demonstrated significant improvement in OSDI scores (−19.4 ± 15.9; *p* < 0.0001), a trend toward improvement in DEQ-5 scores (−1.9 ± 5.5; *p* = 0.059), and a reduction in the meibography score of the inferior eyelid (−0.24 ± 0.60; *p* = 0.023). Intergroup comparisons showed significantly greater improvements in both OSDI and DEQ-5 scores in patients receiving treatment for the ocular surface. Multivariate regression analysis confirmed the association between the use of wipes and tear substitutes and improvements in OSDI (*p* = 0.010) and DEQ-5 scores (*p* = 0.015). No significant postoperative changes in objective OSD parameters were observed in either group. **Conclusions:** The addition of lid wipes and tear substitutes to the standard postoperative regimen significantly improved patient-reported symptoms of ocular discomfort after cataract surgery, while no significant changes were observed in objective signs of ocular surface disease. These findings support the routine use of lid wipes and tear substitutes as an effective strategy for managing postoperative ocular surface discomfort.

## 1. Introduction

Cataract surgery is the most frequently performed surgical procedure worldwide, able to significantly improve patients’ socioeconomic well-being and quality of life. Despite the high success rate, cataract surgery is not without risks, and even uneventful surgeries can lead to undesired effects [1,2]. Ocular surface disease (OSD) is among the most common post-surgical patients’ reported complaints and can be accompanied by symptoms like foreign body sensation, photophobia, burning, fluctuating vision, and epiphora [3].

In recent years, the condition of the ocular surface has been recognized as a key determinant of postoperative visual quality and overall patient satisfaction. An intact and stable tear film is essential for maintaining optical clarity, and even minor surface irregularities can significantly degrade visual function, especially in patients receiving premium intraocular lenses [4,5]. Consequently, increasing attention has been directed toward optimizing ocular surface health before and after cataract surgery as part of the broader concept of patient-centered visual rehabilitation [6].

Several preoperative, intraoperative and postoperative factors can contribute to the onset or worsening of OSD after surgery. First, preoperative dry eye disease (DED), often undiagnosed, may determine higher risks of postoperative worsening [6,7,8]. The prevalence of subclinical DED in cataract candidates has been reported to be remarkably high, and routine preoperative screening is frequently overlooked in standard surgical planning [9,10,11]. Identifying tear film instability, meibomian gland dysfunction, or corneal surface irregularities prior to surgery may allow clinicians to optimize ocular surface conditions and thereby improve postoperative visual outcomes [6]. Secondly, cataract surgery itself may contribute to postoperative DED through factors such as traumatic lid speculum, prolonged exposure to operating microscope light, corneal incisions and consequent nerve damage [12]. Each of these intraoperative stressors may transiently or permanently alter corneal sensitivity and disrupt the neural feedback loop responsible for tear secretion. Furthermore, the duration of surgery, the use of femtosecond laser technology, and the application of high-intensity microscope illumination have been shown to exacerbate postoperative tear film instability [13,14]. Lastly, perioperative topical medications, including antiseptic, antibiotic, and anti-inflammatory eye drops, may damage goblet cells and reduce the quality and quantity of the tear film, especially if preserved [15,16]. Preservatives such as benzalkonium chloride (BAK) can induce epithelial toxicity, meibomian gland dysfunction, and chronic ocular surface inflammation, emphasizing the importance of preservative-free formulations whenever possible [17,18]. The cumulative impact of these multifactorial insults underscores the need for comprehensive perioperative management aimed at maintaining ocular surface integrity and minimizing the risk of postoperative OSD [6].

It is estimated that approximately 37% of patients without pre-existing DED develop it after phacoemulsification cataract surgery [19]. Post-surgical OSD has been shown to significantly impact patients’ satisfaction [4], and although high-performance cataract surgery has achieved excellent refractive outcomes due to advancements in surgical techniques and technology, 20–35% of patients remain dissatisfied despite achieving full vision (unhappy 20/20 patients) [20]. Post-surgical ocular discomfort symptoms can develop as early as one week after surgery and may persist for up to six months, with slow improvement over time [10].

Early recognition and proactive management of postoperative OSD are therefore essential to optimize both functional and subjective outcomes. Several studies have highlighted the benefits of tailored perioperative regimens, including preoperative surface preparation, the use of preservative-free formulations, and postoperative tear supplementation, in reducing the incidence and severity of ocular discomfort [21,22,23]. Moreover, integrating ocular surface assessment into the routine preoperative evaluation has been proposed as a standard of care to enhance refractive predictability and patient satisfaction [6].

Management of postoperative OSD remains challenging, and optimizing its management in the perioperative period is key to improving patient satisfaction and surgical outcomes [24]. First-line treatment for DED typically involves the use of tear substitutes and eyelid hygiene measures to reduce inflammation and improve the overall ocular surface health [24].

The purpose of this study was to evaluate whether the addition of lid wipes and tear substitutes to standard postoperative therapy could improve symptoms and signs of OSD in the early postoperative period in patients undergoing cataract surgery.

## 2. Materials and Methods

This is a retrospective study conducted at the University Hospital of Rome Tor Vergata on adult patients (≥55 years) who underwent routine senile cataract surgery and whose data on ocular surface status before (within 1 week) and 1 month after surgery were available. Patients were excluded if they had a history of contact lens wearing, autoimmune diseases, systemic treatments or conditions known to cause DED, or any prior ocular surgery in the included eye. Additional exclusion criteria included any past or active ocular surface or corneal disease, such as neurotrophic keratopathy, cicatricial conjunctivitis, ocular surface burns, ocular trauma, keratinization of the eyelid margin, or Sjögren’s syndrome.

Symptoms of ocular discomfort were assessed through both the ocular surface disease index (OSDI) and the 5-item Dry Eye Questionnaire (DEQ-5) administered in random order. The OSDI consists of 12 self-administered items covering three main domains: ocular discomfort symptoms (such as light sensitivity, burning, or blurred vision), limitations in visual activities of daily life (including reading, driving at night, or computer use), and the effect of environmental factors like wind or air conditioning. Scores range from 0 to 100, with higher scores reflecting greater symptom severity. The DEQ-5 includes five questions evaluating the frequency and intensity of dryness, discomfort, and tearing experienced over the previous month, with total scores ranging from 0 to 22. An OSDI score of ≥13 and a DEQ-5 score of ≥6 were used to define symptomatic patients [25,26].

The Oculus Keratograph (K5M; Oculus GmbH, Wetzlar, Germany) was used for noninvasive ocular surface examination. This device uses infrared illumination and Placido rings to capture detailed images and assess multiple tear film and ocular surface parameters. Tear meniscus height (TMH) was measured along the central lower lid margin in millimeters using the device’s digital caliper. Noninvasive keratograph tear film break-up time (NIKBUT), including both the ‘first’ break-up time and the ‘average’ break-up time, was automatically determined by the instrument. The first NIKBUT is defined as the interval (in seconds) from the last complete blink to the first distortion of the reflected Placido rings, while the average NIKBUT is the mean of all detected break-up events recorded during the measurement period (up to ~25 s) across the analyzed corneal areas. Infrared meibography of the superior and inferior eyelids was performed to assess meibomian gland loss. Gland loss was graded on a four-point scale, with the device also providing continuous decimal measurements of the percentage of gland loss for more precise quantification. Conjunctival hyperemia was quantified by analyzing anterior segment images and calculating a global redness score based on the density of visible conjunctival vessels.

Snellen’s best-corrected visual acuity (BCVA) was measured in LogMAR. Demographic data, including age, sex, and clinical and ocular history, were recorded from patients’ charts. Operative records and patients’ charts were also used to collect data on postoperative treatment regimens and postoperative ophthalmological assessments.

Cataract surgery was performed under topical anesthesia. The procedure involved a 2.75 mm clear corneal incision, injection of an ophthalmic viscosurgical device (OVD), capsulorhexis, phacoemulsification and bimanual irrigation/aspiration of cortical material. Following implantation of the intraocular lens and removal of the OVD, the incision was routinely hydrated to ensure wound sealing. All procedures were performed by two experienced surgeons, with no significant differences in phacoemulsification technique or surgery duration.

Postoperatively, based on the surgeon’s preference, patients received either standard treatment, consisting of Chloramphenicol 0.5% + Betamethasone 0.2% eye drops (Betabioptal^®^ ophthalmic suspension, Théa Farma S.p.A., Clermont-Ferrand, France) administered four times daily for two weeks, and Bromfenac 0.09% eye drops (Yellox^®^, Bausch & Lomb IOM S.p.A., Bridgewater, NJ, USA) administered twice daily for four weeks, or the same regimen supplemented with lid wipes (Leniva Bio^®^ Lid Wipes, Off Health, Florence, Italy) used twice daily for one week, and tear substitutes (Trimix^®^ eye drops, Off Health, Florence, Italy) used three times daily for three months.

The primary outcome was to evaluate the effect of postoperative lid wipes and tear substitutes on ocular discomfort symptoms one month after cataract surgery. The secondary outcome was to assess their role in reducing clinical signs of ocular surface disease. The study was conducted in accordance with the Declaration of Helsinki and approved by Lazio Area 2 Regional Ethics Committee on 22 May 2025 (Approval ID: 138.25).

### Statistical Analysis

Data are presented as the mean ± standard deviation (SD) or as percentages for categorical variables. T-test and paired *t*-test were used to compare data between groups and between timepoints within the same treatment groups, respectively. Fisher’s exact test was used for 2 × 2 contingency tables. Linear regression was run to evaluate the role of postoperative treatment, age, sex and change in BCVA on DED symptoms as measured by the OSDI and the DEQ-5. All statistical analyses were performed using STATA 18.0 (StataCorp, College Station, TX, USA), and a *p*-value of less than 0.05 was considered statistically significant.

## 3. Results

A total of 63 eyes from 63 patients were included in the analyses. The mean age was 75.1 ± 6.3 years, with 32 patients (51%) being female. Surgery was performed in the right eye in 66.7% of cases. Postoperatively, 23 patients received standard treatment, and 40 received standard treatment plus wipes and tears. Preoperative data for the overall population and for each treatment group are summarized in Table 1. The mean preoperative BCVA was 0.3 ± 0.3 logMAR. Based on symptom questionnaires, DED was identified in 55.1% of patients according to the DEQ-5 and in 85.7% according to the OSDI, with a statistically significant difference between the two tools (*p* = 0.036). The first NIKBUT was below 10 s in 67.3% of patients, while TMH was reduced (<0.20 mm) in 8.1% of cases.

One month after surgery, the mean BCVA improved significantly to 0.04 ± 0.06 logMAR across all groups (*p* < 0.01). No signs of intraocular inflammation or corneal edema/subedema were observed in any patient. In the overall study population, a significant reduction in OSDI scores was observed (−13.0 ± 23.8; *p* = 0.0059), while changes in DEQ-5 scores were not statistically significant (−0.6 ± 5.7; *p* = 0.443).

When analyzed by treatment group, a significant improvement in OSDI scores was found only in patients receiving standard treatment plus wipes and tears (*p* < 0.0001), with no change observed in the standard treatment group (*p* = 0.859) (Table 2). A similar trend was seen for DEQ-5 scores, with a borderline improvement in the combination group (*p* = 0.059) and no significant change in the standard treatment group (*p* = 0.204).

**Figure 1:**
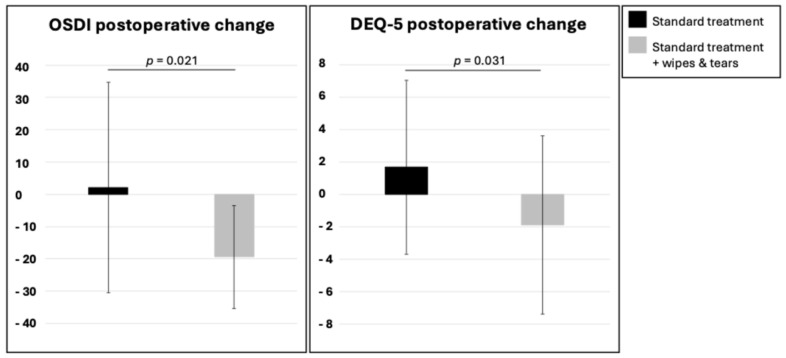
Postoperative changes in OSDI and DEQ-5 in the standard treatment group and in the standard treatment plus lid wipes and tear substitutes group.

Importantly, intergroup comparisons revealed that changes in both OSDI and DEQ-5 scores were significantly greater in patients receiving lid wipes and tear substitutes, favoring their use as an adjunct to standard postoperative therapy (*p* = 0.019 and *p* = 0.032, respectively) (Figure 1).

Patients more symptomatic at baseline also showed to remain more symptomatic after surgery, with positive correlations between preoperative and postoperative values in both the OSDI (ρ: 0.35; *p* = 0.058) and the DEQ-5 (ρ: 0.42; *p* = 0.0027).

No significant postoperative change was observed in any of the ocular surface parameters evaluated, except for a significant improvement in infrared meibography of the inferior eyelid among patients treated with standard therapy plus wipes and tears (−0.24 ± 0.60; *p* = 0.023), compared to a non-significant change in the standard treatment group (+0.17 ± 0.65; *p* = 0.243). Although the direction of change differed between groups, this finding should be interpreted with caution due to baseline differences in inferior meibography scores. Patients in the combination therapy group started with worse baseline values, which may have influenced the observed degree of postoperative improvement (Table 2).

Linear regression confirmed that lid wipes and tear substitutes added to standard treatment significantly ameliorated postoperative DED symptoms (*p* = 0.015 for the DEQ-5 and *p* = 0.010 for the OSDI), while female sex negatively influenced DEQ-5 improvements (*p* = 0.014) (Table 3). Additional analyses controlling for the different baseline values of infrared meibography of the inferior eyelid also confirmed that the addition of wipes and tears was associated with DEQ-5 and OSDI improvements (*p* = 0.010 and *p* = 0.027), that female sex negatively influenced DEQ-5 improvements (*p* = 0.014), and that the baseline value of infrared meibography of the inferior eyelid had no impact on symptom change (*p* = 0.215 and *p* = 0.831 for the DEQ-5 and OSDI, respectively).

## 4. Discussion

This study investigated the impact of postoperative use of lid wipes and tear substitutes on symptoms and signs of OSD after cataract surgery. Our findings demonstrate that initiating lid hygiene and tear supplementation from the day of surgery significantly improves postoperative ocular discomfort compared to standard treatment alone. At one month postoperatively, only patients receiving the combined regimen showed a significant reduction in the OSDI scores and a borderline improvement in DEQ-5 scores. In contrast, patients treated with standard postoperative therapy alone showed no meaningful change in either symptom score, with both questionnaires remaining elevated.

Previous studies have explored the efficacy of various regimens in the perioperative or postoperative management of OSD, including formulations containing hyaluronic acid/trehalose [27], carbomer sodium hyaluronate trehalose and sodium hyaluronate [28], diquafosol [29,30], hydroxypropyl (HP) guar with hyaluronic acid [21], and cyclosporine 0.05% [31].

Among tear substitutes used postoperatively, sodium hyaluronate demonstrated significant benefits, improving symptom scores, tear break-up time (TBUT), and Schirmer test results, reducing epithelial damage, and increasing central corneal epithelial thickness [32,33]. Sodium hyaluronate 0.1% and carboxymethylcellulose 0.5% were shown to enhance TBUT and OSDI scores, while preservative-free HP-Guar formulations alleviate both signs and symptoms of DED and positively modulate inflammatory markers [34]. Additionally, eye drops containing hyaluronic acid and ginkgo biloba increased TBUT at 1 and 4 weeks postoperatively, improved OSDI scores and reduced conjunctival hyperemia at 4 weeks [35]. Sodium hyaluronate with dexpanthenol also demonstrated efficacy in preventing postoperative ocular surface dysfunction at 5 weeks after surgery [36]. Notably, Bharucha et al. reported that postoperative tear substitute use improved patient satisfaction with both vision and surgical outcomes [37].

The inclusion of multiple active ingredients in lubricating eye drops is aimed at targeting various components of the DED vicious cycle, thereby providing broader therapeutic benefits and enhancing patient adherence. In our study, we utilized a multi-action ophthalmic solution containing cross-linked hyaluronic acid 0.15%, trehalose 3%, liposomes 1%, and sterylamine 0.25%. Hyaluronic acid contributes to tear film thickness, stabilizes the mucoaqueous layer, improves lubrication, and supports epithelial cell function through receptor-mediated mechanisms [38,39,40]. Trehalose, a disaccharide with antioxidant, anti-inflammatory, osmoprotective, and cytoprotective properties, supports tear film homeostasis [41,42,43]. Liposomes, consisting of a lipid bilayer encapsulating an aqueous core, act as effective drug carriers and enhance ocular surface lubrication by anchoring the lipid layer to the aqueous component of the tear film [44,45]. Sterylamine, a cationic lipid, promotes liposome adherence to mucins via electrostatic interactions, improving retention, mucoadhesion, bioavailability, and tear film stability while also exerting anti-inflammatory effects [46]. This formulation has previously demonstrated safety and tolerability in patients with moderate to severe DED, producing significant improvements in both signs and symptoms after two months of treatment [47]. Consistent with these findings, our study showed its effectiveness in alleviating postoperative ocular discomfort following cataract surgery.

Eyelid hygiene was also an integral component of our postoperative regimen. Maintaining proper eyelid hygiene in the setting of cataract surgery is crucial for alleviating signs and symptoms of OSD. Eom et al. demonstrated that implementing eyelid hygiene both pre- and postoperatively improved subjective ocular discomfort symptoms and prevented deterioration of meibomian gland expressibility and secretion scores [48]. Similarly, Peral et al. reported that preoperative eyelid hygiene significantly reduced the microbial load on the eyelids [49]. Moreover, a randomized controlled trial found that postoperative eyelid hygiene positively influenced the visual maintenance ratio of functional visual acuity [50], a metric used to evaluate the stability of visual acuity over time, which typically declines in ocular surface disorders such as DED [51]. In our study, lid wipes were used to perform mechanical eyelid hygiene while simultaneously disinfecting the lid margins thanks to lid wipes impregnated with an antiseptic solution containing natural extracts from citrus fruits (Biosecur^®^), Aloe vera, and Ruscus aculeatus, which demonstrated antimicrobial properties [52].

It is important to highlight that our study utilized two different questionnaires, the OSDI and the DEQ-5, both of which produced broadly consistent results. The choice to include both instruments was based on the widespread use of the OSDI for evaluating subjective symptoms before and after cataract surgery [34,35,36]. However, a known limitation of the OSDI is its strong reliance on visual function, which can reduce its specificity in patients with impaired vision. Notably, 7 out of 12 OSDI items relate directly to visual tasks, areas frequently compromised in cataract patients. A previous study examining individual OSDI items pre- and post-cataract surgery found that vision-related items improved significantly after surgery, whereas items specifically addressing dry eye symptoms tended to worsen [53]. Reflecting this dynamic, the OSDI in our study showed a more pronounced postoperative improvement compared to the DEQ-5, which reached only borderline significance. Nonetheless, both questionnaires demonstrated clear symptomatic improvement in patients treated with lid wipes and tear substitutes relative to those receiving standard treatment alone.

Another notable finding is the high prevalence of OSD among cataract patients without a prior diagnosis of DED. In our study, over half of the patients reported DED symptoms preoperatively, as indicated by both questionnaires, and approximately 67% exhibited a NIKBUT shorter than 10 s. These results align with previous reports, confirming that undiagnosed OSD is common in the cataract population. [9,10,54,55]

Most objective signs of DED assessed in our study did not show significant changes compared to preoperative values. This may be partly explained by the inclusion of patients without a prior DED diagnosis, who are less likely to experience postoperative ocular surface alterations [7,8]. Furthermore, surgically induced ocular surface changes typically manifest during the early postoperative period, within days to weeks, and tend to improve over time [19]. The absence of early postoperative follow-up visits in our study represents a key limitation, potentially reducing our ability to detect transient but clinically relevant changes and to fully evaluate the impact of lid wipes and tear substitutes on early DED signs. Additionally, preoperative data on cataract severity were not available, which may have influenced surgical parameters such as phacoemulsification energy and duration, potentially affecting postoperative intraocular inflammation and corneal edema. However, at one month postoperatively, no signs of intraocular inflammation or corneal edema were observed, making it unlikely that these factors contributed to the measurements obtained at this time point. Additional limitations include the retrospective design, the relatively small sample size in each treatment group (though comparable to similar studies [28,31,33,34,35,36]), and the concurrent use of lid wipes and tear substitutes, which preclude determination of the individual contribution of each intervention to the observed postoperative outcomes.

Although our study focused on eyelid hygiene and lubrication in the postoperative period, accumulating evidence suggests that these interventions should ideally begin in the preoperative phase several weeks before surgery and continue throughout the entire perioperative period [21,27,56]. Overall, our findings reinforce the beneficial role of lid wipes and tear substitutes immediately after cataract surgery in alleviating ocular discomfort symptoms, supporting their routine use in postoperative care. Nevertheless, further prospective studies are warranted to determine the optimal treatment protocols that best promote ocular surface health and minimize the risk of OSD in patients undergoing cataract surgery.

## Figures and Tables

**Table 1:** Demographics and clinical characteristics of the overall study population and of the two treatment groups before surgery (*t*-test). M: males; F: females; BCVA: best-corrected visual acuity; OSDI: surface disease index; DEQ-5: 5-item Dry Eye Questionnaire; TMH: tear meniscus height; NIKBUT: noninvasive keratograph break-up time; TMH: tear meniscus height. * significant *p*-value.

Parameter	Overall (n = 63)	Standard Treatment (n = 23)	Standard Treatment + Wipes and Tears (n = 40)	*p*-Value
Age (years)	75.1 ± 6.3	73.4 ± 6.6	76.0 ± 5.9	0.114
Sex (M/F)	31/32	9/14	22/18	0.225
BCVA (logMAR)	0.3 ± 0.3	0.3 ± 0.4	0.3 ± 0.2	0.494
OSDI	34.1 ± 21.1	33.5 ± 21.9	34.3 ± 20.1	0.903
DEQ-5	6.3 ± 6.1	5.6 ± 5.4	6.9 ± 6.1	0.416
TMH (mm)	0.43 ± 0.20	0.40 ± 0.16	0.44 ± 0.22	0.449
NIKBUT first (s)	8.4 ± 5.7	8.7 ± 6.4	8.5 ± 5.6	0.935
NIKBUT average (s)	12.1 ± 5.8	13.2 ± 6.3	11.8 ± 5.7	0.422
Conjunctival hyperemia	1.7 ± 0.5	1.8 ± 0.7	1.6 ± 0.4	0.322
Meibography (superior)	1.4 ± 0.5	1.4 ± 0.5	1.4 ± 0.6	0.921
Meibography (inferior)	1.4 ± 0.7	1.2 ± 0.6	1.6 ± 0.7	0.030 *

**Table 2:** Changes in symptoms and signs of ocular surface disease from baseline to 1 month postoperatively in the two treatment groups (BCVA: best-corrected visual acuity; OSDI: ocular surface disease index; DEQ-5: 5-item Dry Eye Questionnaire; TMH: tear meniscus height; NIKBUT: noninvasive keratograph break-up time; TMH: tear meniscus height). * significant *p*-value.

Parameter	Postoperative Change				
	Standard Treatment (n = 23)		Standard Treatment + Wipes and Tears (n = 40)		
	Mean ± SD	*p*-Value (vs. Baseline)	Mean ± SD	*p*-Value (vs. Baseline)	*p*-Value (Between Groups)
BCVA (logMAR)	−0.3 ± 0.4	0.005 *	−0.24 ± 0.16	<0.0001 *	0.423
OSDI	+2 ± 32.7	0.859	−19.4 ± 15.9	<0.0001 *	0.021 *
DEQ-5	+1.7 ± 5.4	0.204	−1.9 ± 5.5	0.059	0.031 *
TMH (mm)	+0.03 ± 0.15	0.288	−0.04 ±0.20	0.212	0.124
NIKBUT first (s)	−2 ± 7.3	0.304	−1.9 ± 7.1	0.150	0.967
NIKBUT mean (s)	−2.25 ± 7.1	0.256	−1.3 ± 7.9	0.348	0.718
Conjunctival hyperemia	+0.09 ± 0.64	0.549	+0.006 ± 0.4	0.933	0.568
Meibography (superior)	+0.07 ± 0.47	0.496	−0.1 ± 0.5	0.229	0.207
Meibography (inferior)	+0.17 ± 0.65	0.243	−0.24 ± 0.60	0.023 *	0.020 *

**Table 3:** Multivariate linear regression. BCVA: best-corrected visual acuity; OSDI: surface disease index; DEQ-5: 5-item Dry Eye Questionnaire; * significant *p*-value.

		Age	Sex (Female = 1)	Wipes and Tears (Yes = 1)	BCVA Improvement
DEQ-5	Coefficient	−0.25	−3.68	+4.0	n/a
improvement	*p*-value	0.068	0.014 *	0.015 *	
OSDI improvement	Coefficient	−0.51	−15.6	+25.0	+4.2
	*p*-value	0.593	0.072	0.010 *	0.755

## Data Availability

Data are available from the corresponding author upon reasonable request.
